# Cell wall bricks of defence: the case study of oligogalacturonides

**DOI:** 10.3389/fpls.2025.1552926

**Published:** 2025-03-25

**Authors:** Chiara Degli Esposti, Laura Guerrisi, Giulia Peruzzi, Sarah Giulietti, Daniela Pontiggia

**Affiliations:** ^1^ Department of Biology and Biotechnologies “Charles Darwin”, Sapienza University of Rome, Rome, Italy; ^2^ Research Center for Applied Sciences for the Protection of the Environment and Cultural Heritage, Sapienza University of Rome, Rome, Italy

**Keywords:** cell wall, DAMP (damage associated molecular pattern), oligogalacturonides (OGs) plant-microbe interactions, biotic stress, plant immunity elicitor, crop protection, sustainable agriculture, eco-friendly agriculture

## Abstract

The plant cell wall (CW) is more than a structural barrier; it serves as the first line of defence against pathogens and environmental stresses. During pathogen attacks or physical damage, fragments of the CW, known as CW-derived Damage-Associated Molecular Patterns (CW-DAMPs), are released. These molecular signals play a critical role in activating the plant’s immune responses. Among CW-DAMPs, oligogalacturonides (OGs), fragments derived from the breakdown of pectin, are some of the most well-studied. This review highlights recent advances in understanding the functional and signalling roles of OGs, beginning with their formation through enzymatic CW degradation during pathogen invasion or mechanical injury. We discuss how OGs perception triggers intracellular signalling pathways that enhance plant defence and regulate interactions with microbes. Given that excessive OG levels can negatively impact growth and development, we also examine the regulatory mechanisms plants use to fine-tune their responses, avoiding immune overactivation or hyper- immunity. As natural immune modulators, OGs (and more generally CW-DAMPs), offer a promising, sustainable alternative to chemical pesticides by enhancing crop resilience without harming the environment. By strengthening plant defences and supporting eco-friendly agricultural practices, OGs hold great potential for advancing resilient and sustainable farming systems.

## From barrier to signal: how the cell wall shapes plant immunity

Plant cells are surrounded by the cell wall (CW), a rigid yet dynamic extracellular matrix that provides biomechanical support and regulates various physiological processes (Keegstra, 2010). The CW plays a fundamental role in determining cell size and shape, supporting mechanical stability, and mediating cell expansion, adhesion, and communication (Alonso Baez and Bacete, 2023). To fulfil these functions, the CW undergoes continuous remodelling during plant growth and development (Lorrai and Ferrari, 2021).

As the primary interface between the cell and its external environment, the CW is frequently exposed to biotic and abiotic stresses, which can lead to structural damage (Alonso Baez and Bacete, 2023; Geitmann, 2024). Consequently, plant cells have evolved sophisticated mechanisms to monitor CW integrity and maintain homeostasis under environmental challenges (Lee and Santiago, 2023; Geitmann, 2024). Although the regulatory mechanisms controlling CW composition and structure are not yet fully understood, it is well-established that feedback processes coordinate CW-derived signals with specific intracellular responses (Wolf, 2022). Recent advances in this field have revealed a complex and highly integrated system that continuously monitors the chemo-mechanical properties of the CW and triggers restorative or compensatory responses when necessary (Munzert and Engelsdorf, 2024). Additionally, CW damage caused by biotic and abiotic stresses must be perceived as a danger signal to activate rapid and efficient defence mechanisms (Savatin et al., 2014b; Lorrai and Ferrari, 2021; Pontiggia et al., 2024).

Every plant cell has the capacity to mount a complex and advanced innate immune response to defend itself against pathogens and pest attacks. This immune activation relies on the cell’s ability to detect and interpret danger signals accurately. Plant immunity operates through two layers of defence: pattern-triggered immunity (PTI) and effector-triggered immunity (ETI) (Jones and Dangl, 2006). According to the classical “zig zag model”, these two responses were historically considered distinct. However, emerging evidence demonstrate their strict interdependence in orchestrating an effective immune response (Yuan et al., 2021; Ngou et al., 2022).

In PTI, danger signal perception typically occurs at the cell surface through a wide-range of membrane-localised receptors, called Pattern Recognition Receptors (PRRs) (Bender and Zipfel, 2023). PRRs can efficiently recognise both “non-self” danger signals, known as Microbe-Associated Molecular Patterns (MAMPs), and “self” signals, referred to as Damage-Associated Molecular Patterns (DAMPs) (Tanaka and Heil, 2021; De Lorenzo and Cervone, 2022). MAMPs are conserved microbial molecules such as bacterial flagellin and fungal chitooligomers, while DAMPs are endogenous molecules produced and released upon cell injury caused by mechanical damage or pathogen attack (Tanaka and Heil, 2021; De Lorenzo and Cervone, 2022).

While PTI is regarded as the basal, first-line defence response, Effector-Triggered Immunity (ETI) is activated by microbial effectors released into the plant apoplast or cytoplasm and detected by specific plant resistance (R) proteins (DeFalco and Zipfel, 2021). ETI is characterised by a more robust and prolonged response compared to PTI, often culminating in localised programmed cell death known as the “hypersensitive response” (HR) (Ngou et al., 2022). Recent studies have shown that PTI and ETI act synergistically to enhance disease resistance (Yuan et al., 2021; Ngou et al., 2022). Additionally, the local perception of danger signals initiates systemic immune responses in distal tissues, mediated by defence-related hormones such as salicylic acid (SA) or jasmonic acid (JA), which play distinct yet complementary roles (Vega-Munoz et al., 2020; Kim and Lim, 2023).

It is not surprising that the CW, as the external interface and primary defence against pathogens, generates diverse types of DAMPs when fragmented (Bacete et al., 2018; Pontiggia et al., 2020; De Lorenzo and Cervone, 2022). The ability of plants to recognise CW fragments as immunogenic molecules likely has an evolutionary ancient origin and can be considered a primitive form of pathogen perception (Pontiggia et al., 2020; De Lorenzo and Cervone, 2022).

## Plant cell wall-degrading enzymes

In nature, plants are colonised by diverse microbial communities. These microorganisms can behave in different ways, often shifting roles: some act as pathogens and cause diseases, others coexist without harming the plant, while some are symbionts, establishing mutually beneficial relationships (Hassani et al., 2018).

During colonisation, bacteria and fungi release a variety of cell wall-degrading enzymes (CWDEs) that break down the plant CW. These enzymes help degrade the main structural components of the wall, releasing sugars that microbes use for nutrition and survival (Kubicek et al., 2014; De Lorenzo and Cervone, 2022; Wang et al., 2022). Different types of CWDE perform specific functions, including the hydrolysis of glycan linkages, oxidation or reduction of glycans, and modification of polysaccharides (Molina et al., 2024a). This diversity reflects the complex structure of the plant CW, which microbes must overcome to invade plant cells while also generating glycans for their own nutrition. The variety of CWDEs highlights the different strategies used in plant–microbe interactions. Biotrophic and hemi-biotrophic microorganisms, which rely on living host tissues for colonisation, generally have fewer CWDEs in their genomes. Similarly, beneficial microbes or symbionts, such as mycorrhizal fungi, do not require large amounts of CWDEs. Instead, they regulate CW degradation carefully, maintaining limited enzymatic activity (Balestrini and Bonfante, 2014; Redkar et al., 2022). Studies have shown that the transition from a saprotrophic to a symbiotic lifestyle often involves the loss of CWDEs in fungal genomes, demonstrating that symbiosis is compatible with reduced CWDE activity (Kohler et al., 2015; Miyauchi et al., 2020; Mesny et al., 2021; Pontiggia et al., 2024). This reduction helps minimise damage to plant tissues, allowing fungi to sustain their lifecycle and strengthen interactions with the host. For instance, polygalacturonases (PGs) produced by beneficial endophytic *Trichoderma* spp. are essential for establishing symbiosis (Moran-Diez et al., 2009). Recent studies indicate that *Bacillus velezensis* can sense pectic homogalacturonan and OGs, which are released from plant CW during microbial colonisation. The bacterium employs specific pectinolytic enzymes, such as PGs, to degrade pectin, enhancing its establishment and persistence in the rhizosphere. This interaction between *Bacillus* and plant-derived pectin fragments plays a crucial role in facilitating successful rhizosphere colonisation (Boubsi et al., 2023; Kovacs, 2024). Also, PGs facilitate smooth root penetration and play a key role in priming Induced Systemic Resistance (ISR) (Redkar et al., 2022). In contrast, necrotrophic pathogens, which thrive on dead tissue, possess a higher number of CWDEs, such as cellulases, pectinases, and xylanases. This allows them to aggressively degrade plant cell walls, leading to significant tissue damage (Kubicek et al., 2014; Mesny et al., 2021). The evolutionary variation in CWDE profiles highlights the connection between pathogen lifestyle and the structural complexity of plant cell walls. This reflects the continuous arms race between plant defences and microbial strategies for invasion and colonisation.

## Plant cell wall as a source of damage-associated molecular patterns

Plants have evolved precise and effective systems to detect perturbations in the CW structure and the activity of CWDEs by sensing their products (Pontiggia et al., 2024). Initially, danger signals released from the CW were referred to as “endogenous elicitors” (Hahn, 1981). However, these signals are more accurately described as Damage-Associated Molecular Patterns (DAMPs), specifically CW-DAMPs, which play a crucial role in plant immunity (Hou et al., 2019; Wan et al., 2021; Molina et al., 2024a). The structural and biochemical complexity of the CW, combined with the diverse range of CWDEs used by microbes during plant interactions, generates various CW fragments that are recognised by host cells as signalling molecules (Molina et al., 2024b).

The CW primarily consists of carbohydrates and phenolic compounds, with a small proportion of structural proteins (Geitmann, 2024). Cellulose, the most abundant polymer in the CW, is composed of linear, unbranched β-(1,4)-linked glucan chains that are highly ordered and rigid (Zhang et al., 2021). The matrix includes hemicelluloses, represented by xyloglucans, glucomannans, heteroxylans, mixed-linkage glucans (MLGs), and pectins (Scheller and Ulvskov, 2010). Hemicelluloses, more structurally diverse than cellulose, are polysaccharides mainly characterised by β-1,4-linked backbone, except for MLGs – found only in *Poaceae* – which feature both β-1,4 and β-1,3 linkages (Yang et al., 2021a). Pectins, predominantly present in primary CW, have a backbone of α-(1,4)-linked galacturonic acids and are categorised into homogalacturonan (HG), rhamnogalacturonan I (RG-I), and the highly branched rhamnogalacturonan II (RG-II) (Zhang et al., 2021; Munzert and Engelsdorf, 2024). The HG backbone can be methyl-esterified, influencing the formation of calcium-mediated crosslinks. RG-I, composed of repeated dimeric units, is heavily acetylated and branched with arabinans, galactans, or arabinogalactans. RG-II is the most complex pectin structure, containing 12 different sugars and numerous linkages, further modified by methylation and acetylation (Mohnen, 2008; Wachananawat et al., 2020; Begum and Fry, 2023).

Over the past decade, numerous CW fragments with plant immunity-inducing properties have been identified (Molina et al., 2024a). For example, cellulose degradation generates cellodextrins (CDs), which have demonstrated immune-eliciting activity in various plant species (Aziz et al., 2007; Souza et al., 2017; Johnson et al., 2018; Locci et al., 2019; Costantini et al., 2024). Fragments of hemicellulose, such as mannan-, xyloglucan-, and arabinoxylan-derived oligosaccharides, also function as CW-DAMPs (Claverie et al., 2018; Heloir et al., 2019; Zang et al., 2019; Mélida et al., 2020; Molina et al., 2024b). Furthermore, fragments of MLGs elicit immune responses in both monocots and dicots plants (Barghahn et al., 2021; Rebaque et al., 2021; Yang et al., 2021a; Costantini et al., 2024). However, because MLGs are only present in the CW of *Poaceae* and microbial organisms (Gigli-Bisceglia et al., 2020; Yang et al., 2021a), they act as typical MAMPs in most other plant species (Barghahn et al., 2021; Rebaque et al., 2021; Yang et al., 2021a).

Recently, oligomers derived from the fragmentation of the cuticle a hydrophobic protective barrier composed of a cutin matrix integrated with waxes have been shown to act as plant immunity elicitors (Moreira et al., 2024). Linear β-1,3-glucans, another class of CW fragments with elicitor activity can originate from both pathogens and plants, functioning either as MAMPs or DAMPs, respectively (Voigt, 2014; Mélida et al., 2018; Wanke et al., 2020). As MAMPs, they are components of fungal CW (Mélida et al., 2018; Wanke et al., 2020). As DAMPs, linear β-1,3-glucan fragments are released from callose within plant papillae as result of pathogen degrading enzyme activity (Voigt, 2014; Mélida et al., 2018).

**Figure 1:**
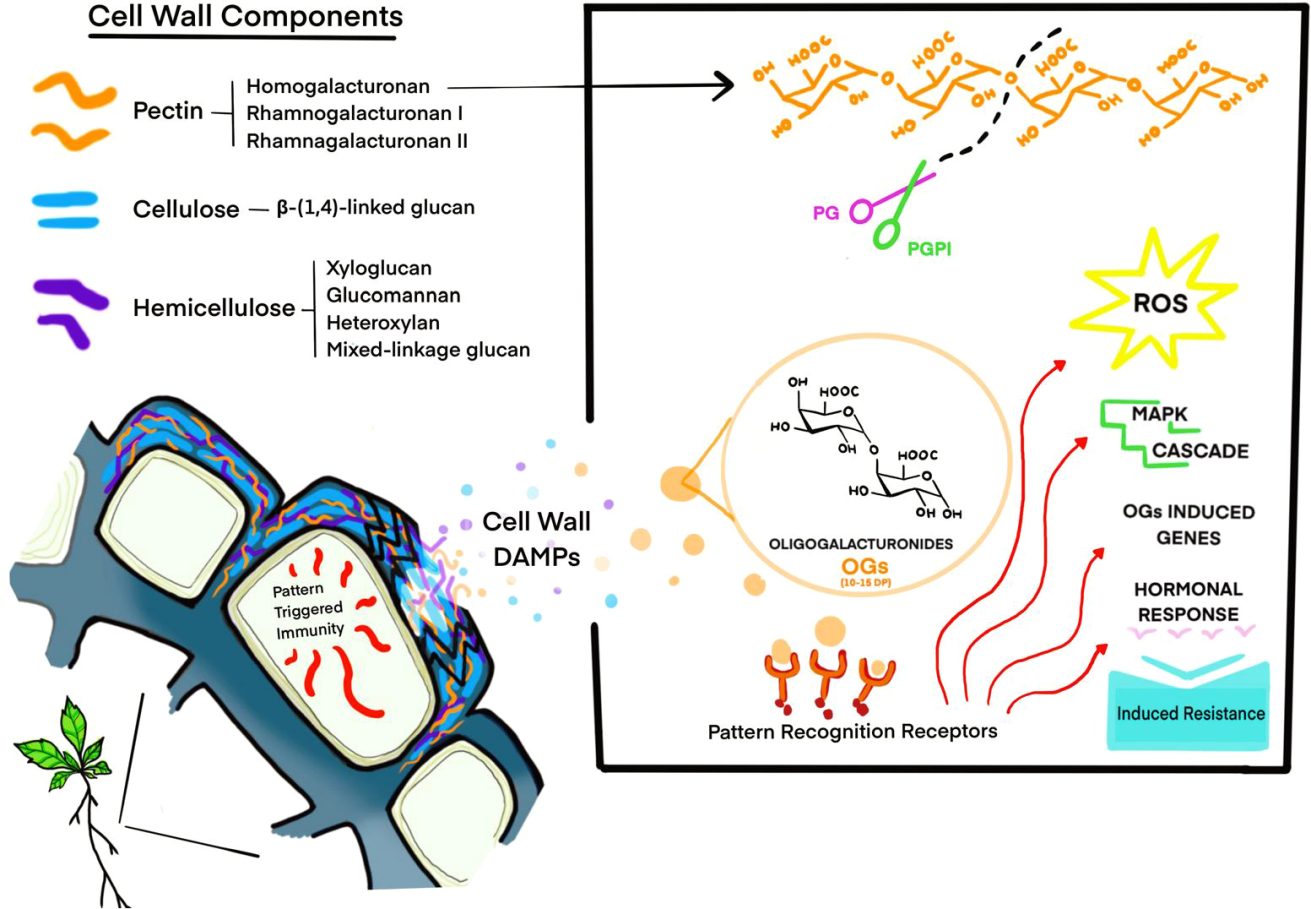
Plant cell wall structure and oligogalacturonides (OGs) signalling pathway. The illustration represents the plant cell wall and the release of DAMPs, such as OGs, upon CW rupture. The inset illustrates OG biosynthesis: homogalacturonan is cleaved by PG, whose action is modulated by PGIP to produce and accumulate OGs with a DP of 10–15. OGs are recognised by Pattern Recognition Receptors (PRRs) on the cell surface, triggering signalling cascades that involve Reactive Oxygen Species (ROS) production, activation of Mitogen-Activated Protein Kinase (MAPK) pathways, transcriptional reprogramming and hormone production and accumulation. Together, these responses collectively enhance plant resistance to pathogens.

The best-characterised class of CW-DAMPs are oligogalacturonides (OGs). Bioactive OGs, fragments of α-(1,4)-linked d-galacturonic acid with a degree of polymerisation (DP) of 10-15 residues, are capable of eliciting immune responses upon recognition by plant cells (Ferrari et al., 2013). Interestingly, OGs exhibit a dose-dependent effect: at high concentrations they induce immune responses, while at lower concentrations they regulate plant growth and development (Ferrari et al., 2013; Pontiggia et al., 2020; Yang et al., 2021c) (**Figure 1**).

CW fragmentation is not solely a consequence of microbial degradation but also occurs during plant CW remodelling in physiological processes (De Lorenzo and Cervone, 2022). To maintain CW-DAMPs homeostasis and prevent hyper-immunity, which could strongly impact fitness and survival, plants have developed mechanisms to regulate their accumulation (Pontiggia et al., 2020).

## The PG-PGIP interaction: a mechanism that releases oligogalacturonides to activate plant immunity

The immune role of OGs in plants was initially characterised trough biochemical studies on fungal endo-polygalacturonase (PG) activities, which act as pathogenic factors (Pontiggia et al., 2024). These studies revealed how plants evolved a defence system to counteract the effects of PGs by producing inhibitors called polygalacturonase-inhibiting proteins (PGIPs). PGIPs are highly specific, targeting particular types of PGs (De Lorenzo et al., 2001; D'Ovidio et al., 2004b).

PGIPs can significantly reduce the efficiency of fungal PGs, thereby slowing down pathogen progression, limiting the degradation of HG and extending the half-life of OGs (D'Ovidio et al., 2004a; Kalunke et al., 2015). Recent studies have shown that some fungal pathogens produce extracellular effectors capable of inactivating PGIPs (Wei et al., 2022). This discovery highlights the importance of PGIPs in defence.

The interaction between the PG from *Fusarium phyllophorum* and the PGIP from *Phaseolus vulgaris* has been well studied for many years (D'Ovidio et al., 2004b; Di Matteo et al., 2006; Federici et al., 2006; Casasoli et al., 2009; Benedetti et al., 2013). Recently this interaction recently led to the resolution of the crystallographic structure of the complex PG-PGIP (Xiao et al., 2024). Structural analysis has confirmed that plant PGIPs not only regulate PG activity but also convert this virulence factor into an enzyme that triggers defence responses by promoting the production and accumulation of OGs with a degree of polymerisation (DP) between 10 and 15 (Mooney and van der Hoorn, 2024; Xiao et al., 2024). Interestingly, PvPGIP2 does not block the active site of *Fp*PG directly: rather, it remodels it by creating a tunnel in PvPGIP2 that includes additional substrate-binding regions (Thynne and Kobe, 2024).

## Defence activation through OG perception: from early signals to resistance mechanisms

Despite extensive research on OGs and their biological responses, the precise mechanism of their recognition remains unresolved. Wall-associated kinases (WAKs) are implicated in sensing CW integrity (Kohorn, 2016), with WAK1 in *Arabidopsis* proposed as a receptor for OGs (Brutus et al., 2010). However, studies using mutants with silenced or deleted WAK family members (Herold et al., 2024) have produced contradictory results, suggesting that OG perception may involve multiple, partially redundant perception/transduction complexes (Gravino et al., 2017). It is undeniable that WAKs are key components of the CW-mediated signalling in the plant immune system, actively participating in pathogen recognition and activation of defence responses (Stephens et al., 2022). The notion that WAKs may be dispensable for several OG activities aligns with the understanding that OGs exert pleiotropic effects in both immunity and development, influencing various biological and physiological processes in a complex, multifaceted manner (Ferrari et al., 2013; Gravino et al., 2017). This suggests that OGs can trigger immune and growth responses through alternative mechanisms, while WAKs may not necessarily be involved in all OG-mediated events but still contribute to a broader regulation of the plant overall responses.

Along these lines, Blaschek (2024) proposes several models for WAK function, summarised in **Table 1**, to address discrepancies between recent findings and prior evidence: one model suggests that WAKs act as OGs receptors but, when disrupted, are functionally compensated by WAK-like or analogous receptors; another proposes that WAKs detect pathogen-derived molecules; and a third model indicates that WAKs sense the physical connection between the plasma membrane and the CW, possibly via an apoplastic partner analogous to FERONIA and Leucine-rich Repeat Extensins interaction (Blaschek, 2024). Recent evidence (Liu et al., 2024) thus indicates that de-esterified OGs can bind Rapid-Alkalinization-Factors (RALFs) peptides, particularly RALF1 and RALF23, forming particles in the apoplast through a process known as liquid-liquid phase separation. These particles directly interact with the FERONIA-LLG1 membrane complex, triggering the clustering of various cell surface regulators such as BRASSINOSTEROID INSENSITIVE 1 (BRI1) and FLAGELLIN-SENSITIVE 2 (FLS2). This clustering initiates a process of widespread and promiscuous endocytosis, during which these regulators are removed from the plasma membrane, although OGs-RALFs particles remain in the apoplast. This endocytic mechanism plays a role in plant strategies for mitigating abiotic stress, such as salt and heat stress. Liu et al. (2024) also demonstrated that knock-out mutants for FERONIA, its coreceptor LLG1, and the RALF1 peptide show reduced responses to OGs, particularly in ROS production and the induction of endocytosis. Similarly, a recent study by Giovannoni et al. (2024) revealed that mutants with impaired endocytosis exhibit defects in typical OG-triggered responses, including extracellular alkalinisation, MAPK phosphorylation, and early defence gene activation. Although the specific role of this process in OG recognition and signalling pathways remains unclear, these findings combined with conflicting evidence on the role of WAKs (Herold et al., 2024; Kohorn et al., 2021; Liu et al., 2023) suggest that OG perception may involve multiple redundant mechanisms rather than a single receptor.

**Table 1:** Evidence of WAKs perception models.

MODEL	SUPPORTING EVIDENCE	REFERENCE
WAKs are OG receptors, but their loss is functionally compensated by redundant WAK-like or analogous receptors	The response to OGs is activated by different perception/transduction pathways. Interactors of WAKs affect OG perception	Gravino et al., 2017; Gramegna et al., 2016
	FERONIA and WAKs play a role in sensing pectin-derived elicitors of plant defence released upon lignin modifications	Liu et al., 2023
	*wakΔ2* (deletion of WAK1–5) retains full OG responsiveness in both early and late immune signalling responses	Herold et al., 2024
WAKs perceive molecules of microbial origin	WAK3 is required for immune responses activated by a bacterial peptide	Held et al., 2024
WAKs may jointly play with other interactors of OGs in the apoplast	The connection between the plasma membrane and CW occurs through FERONIA and Leucine-rich Repeat Extensins.	Dunser et al., 2019
	OGs bind RALF peptides in the apoplast and activate FERONIA-LLG1 complexes .	Liu et al., 2024

Nevertheless, it is well-established that OGs (in particular with DP 10–15) act as molecular sentinels of CW perturbation, triggering a wide spectrum of plant defence mechanisms that range from immediate intracellular signalling to longer-lasting structural and biochemical reinforcements. OG-induced responses, widely studied across different plant systems, include rapid events such as reactive oxygen species (ROS) production, increases in cytosolic calcium and nitric oxide levels, and the activation of MAPKs and calcium-dependent protein kinases (CDPKs), as summarised in **Table 2** (Bellincampi et al., 2000; Galletti et al., 2008; Giulietti et al., 2023; Rasul et al., 2012; Gravino et al., 2015; Marti et al., 2021). These responses often overlap with MAMP-triggered pathways, such as flg22-induced PTI (Hahn, 1981; Davis and Hahlbrock, 1987; Broekaert and Pneumas, 1988; Denoux et al., 2008; Galletti et al., 2008; Lee et al., 2022). Transcriptomic analysis reveals substantial early overlap between OG- and flg22-induced immune responses, particularly in ROS and MAPKs signalling, which are mediated by AtRBOHD and kinases such as ANPs (Denoux et al., 2008; Galletti et al., 2011; Savatin et al., 2014b). This highlights a shared signalling framework despite differences in oxidative burst roles. For example, while the oxidative burst is essential for callose deposition in both pathways, it is not required for early marker genes activation and OG-induced resistance to *Botrytis cinerea*. In contrast, ROS play a critical role in flg22-mediated resistance to *Pseudomonas syringae* (Zhang et al., 2007). The unique complexity of OG signalling is further highlighted by the partial dependence on leucine-rich repeat co-receptors BAK1/SERK3 and BKK1/SERK4, which are essential for flg22 responses but impact only a subset of OG-induced defences (Gravino et al., 2017). This suggests that specific phosphorylation events fine-tune OG-triggered signalling, resulting in distinct downstream outcomes (Mattei et al., 2016). Regarding the diverse roles of the oxidative burst, it has been shown that ROS production induced by OGs in the symbiotic bacterium *Rhizobium leguminosarum* triggers a calcium response and activates oxidative stress-related genes without compromising bacterial viability (Moscatiello et al., 2012). Additionally, OGs suppress flavonoid-induced expression of nodulation genes, revealing a complex signalling interplay during the early stages of plant-microbe interactions (Moscatiello et al., 2012). These findings help to elucidate the role of OGs in facilitating plant-beneficial microbe interactions.

**Table 2:** Overview of defence responses triggered by OGs across various plant species.

Type of Defence Response	Plant Species and Study System	References
ROS production	*A. thaliana* (leaf)	Gravino et al., 2017; Giovannoni et al., 2021; Silva-Sanzana et al., 2022; Giovannoni et al., 2024
	*A. thaliana* (cotyledons)	Savatin et al., 2014a; Marti et al., 2021
Phytoalexin accumulation	*A. thaliana* (seedlings)	Savatin et al., 2014a
	*Pisum sativum* (root)	Selim et al., 2017
PR-gene expression	*Solanum lycopersicum* (leaf)	van Aubel et al., 2016; Bektas, 2022; Rakoczy-Lelek et al., 2023;
	*Solanum tuberosum (leaf)*	Clinckemaillie et al., 2017
	*Olea europaea* (leaf)	Varveri et al., 2024
	*A. thaliana* (leaf)	Howlader et al., 2020
	*Fragaria vesca (fruit)*	Osorio et al., 2008
Callose deposition	*A. thaliana* (leaf)	Galletti et al., 2008; Gravino et al., 2017; Silva-Sanzana et al., 2022; Salvati et al., 2024a
MAPK signalling	*A. thaliana* (seedlings)	Savatin et al., 2014a; Gravino et al., 2017; Marti et al., 2021
	*Solanum lycopersicum* (leaf)	Rakoczy-Lelek et al., 2023;
Hormonal changes (JA/SA/ET)	*Solanum lycopersicum* (leaf)	van Aubel et al., 2016; Gamir et al., 2021; Bektas, 2022; Rakoczy-Lelek et al., 2023;
	*A. thaliana* (leaf)	Gravino et al., 2015; Silva-Sanzana et al., 2022; Howlader et al., 2020; Liu et al., 2023
	*Fragaria vesca (fruit)*	Osorio et al., 2011
Induction of systemic signals	*Solanum lycopersicum*	Gamir et al., 2021
Stomatal closure	*Olea europaea* (leaf)	Varveri et al., 2024

The table summarises the main activated mechanisms, plant species, and experimental systems studied, based on recent research from the past few years.

Exogenous OG treatments have demonstrated broad-spectrum protective effects against necrotrophic pathogens such as *B. cinerea* and *Pectobacterium carotovorum* (Aziz et al., 2004; Ferrari et al., 2007; Rasul et al., 2012), hemi-biotrophic pathogens like *P. syringae* (Howlader et al., 2020), and even aphids (*Myzus persicae*) (Silva-Sanzana et al., 2022). Additionally, the transgenic expression of a PGIP-PG chimera, known as the “OG-machine” enables the *in vivo* production of endogenous OGs, enhancing resistance in Arabidopsis against *B. cinerea*, *P. carotovorum*, and *P. syringae* (Benedetti et al., 2015). Interestingly, OG-mediated local resistance operates independently of major phytohormone signalling pathways, such as salicylic acid (SA), jasmonic acid (JA) and ethylene (ET). Instead, it relies on PHYTOALEXIN DEFICIENT 3 (PAD3), a cytochrome P450 catalysing the final step of camalexin biosynthesis (Ferrari et al., 2007). Beyond *A. thaliana*, the elicitor activity of OGs has been observed in other plant species. For instance, in wheat, pretreatment with OGs prevents the infection of *Blumeria graminis* f. sp. *tritici* (Randoux et al., 2010) and *Fusarium graminearum* (Bigini et al., 2024). Similarly, OGs provide resistance to *Verticillium dahliae* in cotton and *Rhizoctonia solani* in rice (Wen et al., 2024). In *Pisum sativum*, pretreatment with OGs confer high and stable protection against the oomycete *Aphanomyces euteiches* (Selim et al., 2017). Furthermore, in indoor-grown sugar beet plants, OGs exhibit antimicrobial activity against *R. solani*, triggering defence-gene expression and limiting pathogen spread in plant roots (Zhao et al., 2022).

The widespread presence of OG-induced defence responses across different plant species underscores their potential applications in sustainable agriculture (see specific paragraph below).

## OG-induced hormone signalling

OGs activate multiple plant hormonal pathways, with JA and SA being central to their function. In general, the SA signalling pathway is crucial for defence against biotrophic and hemibiotrophic pathogens, while the ET/JA signalling pathway plays a key role in activating resistance to necrotrophic pathogens and herbivorous insects (Aerts et al., 2021).

OGs upregulate genes involved in both JA and SA pathways, underscoring their ability to orchestrate a broad-spectrum of defence responses (Denoux et al., 2008; Gravino et al., 2015; Liu et al., 2023). Treatments with OGs in *A. thaliana* have been found to up-regulate genes essential for JA biosynthesis and signalling (Simpson et al., 1998; Davidsson et al., 2017; Gamir et al., 2021). Notably, only pectinases activity, not cellulases or xylanases, triggers the JA pathway, suggesting pectin as the primary elicitor for JA-induced responses (Engelsdorf et al., 2018). OGs are critical regulators of SA-mediated immune responses in plants by triggering PTI, which includes the activation of SA biosynthesis and signalling pathways. This immune activation often involves the upregulation of genes such as *ICS1* and *PR1*, markers of SA-mediated defences (Denoux et al., 2008; Gravino et al., 2015). In addition to enhancing pathogen resistance, OGs increase plant defences against herbivores by boosting SA levels. Indeed, treatment with OGs in *A. thaliana* has been shown to elevate SA-associated immune responses, including callose deposition and ROS production, leading to improved resistance against aphid infestations (Silva-Sanzana et al., 2022).

JA plays a vital role in responses to necrotrophic pathogens and mechanical damage, while SA is critical for defence against biotrophic pathogens. OGs activate these pathways synergistically, as seen in resistance to *P. syringae pv. tomato* DC3000, challenging the traditional antagonism between JA and SA signalling (Howlader et al., 2020; Liu et al., 2023). ROS production and calcium signalling further amplify the interplay between these pathways, integrating local and systemic immune responses (Engelsdorf et al., 2018; Tripathi et al., 2018). Moreover, the effects of OGs are modulated by structural features, with DPs 10–15 being the most potent inducers of JA and SA pathways. These findings highlight OGs not only as key initiators of plant defence but also as modulators of hormonal crosstalk, enabling tailored responses to specific threats (Kohorn, 2016; Liu et al., 2023). Actually, the traditional view that JA and SA operate in antagonism has been replaced by a more integrated view of their interactions, where they can both act synergistically and antagonistically depending on the context, ultimately enhancing the plant’s ability to defend itself against a wide range of biotic stresses (Howlader et al., 2020).

OGs also stimulate ethylene production by activating signalling pathways that enhance the activity of biosynthetic enzymes such as like 1-aminocyclopropane-1-carboxylate (ACC) synthase (ACS) and ACC oxidase (ACO) (Zhou et al., 2023). Triggering MAPKs cascade, OGs directly regulate ACS activity *via* phosphorylation, increasing ethylene levels and promoting defence responses (Zhou et al., 2023). Moreover, an intact ethylene signalling is required for OG-induced protection against *B. cinerea* (Gravino et al., 2015).

The perception of OGs not only triggers defence mechanisms but also influences plant growth, particularly through their interaction with auxin, which plays a critical role in regulating various aspects of growth, such as cell elongation, division, and differentiation (Branca et al., 1988; Bellincampi et al., 1993). Perception of OGs inhibits auxin responses in a dose-dependent manner, thereby modulating growth responses (Savatin et al., 2011). OG-induced growth inhibition seems to be promoted also by ethylene production (Zhou et al., 2023). In addition to their inhibitory activity on auxin-dependent signalling, in *A. thaliana* plants expressing the ‘OG-machine’, the production and accumulation of OGs *in vivo* not only confer resistance to pathogens but were associated with severely reduced or completely arrested growth (Benedetti et al., 2015). It should be noted that higher SA levels were detected in these plants following induction of the chimeric protein, suggesting further regulation in the defence growth trade-off dependent on the interaction between OG- and hormone-mediated signalling. Therefore, given their nature as cell wall components, which are closely linked to the processes of cell wall growth and remodelling, and their ability to elicit defence responses, it is reasonable to consider that OGs may act as regulators of the growth-defence trade-off.

## OG-triggered systemic responses

The ability of OGs to move within plant tissues remains questionable, with conflicting evidence reported in the literature (Bishop et al., 1981; Baydoun and Fry, 1985; MacDougall et al., 1992). While local responses to OGs are relatively well understood, systemic responses those occurring in distal sites of the plant remain poorly characterised (Harris and Mou, 2024). However, the well-documented occurrence of systemic defence responses to wounding and pathogens in plants (Hilleary and Gilroy, 2018), raises the possibility that OGs may play a role in the long-distance signalling.

Early studies demonstrated the capacity of OGs to elicit systemic responses in tomato, inducing defence-related processes (Bishop et al., 1981; Reymond et al., 1995; Thain et al., 1995; Simpson et al., 1998). However, evidence for systemic resistance against *B, cinerea* mediated by OGs has been reported only in *A. thaliana* (Ferrari et al., 2007), and the molecular mechanisms underlying this phenomenon remain unexplored. More recently (Gamir et al., 2021), revisited the effects of OGs in tomato, examining spatial and temporal changes in hormone accumulation, gene expression, and metabolome profiles in systemic tissues following OGs application to lower leaves and roots. The study revealed that the abscisic acid (ABA) precursor ABA-glucoside, JA precursor 12-oxo-phytodienoic acid (OPDA), and, to a lesser extent, SA, accumulated in primed leaf tissues, peaking six hours post-treatment. Remarkably, root tissues of plants treated with OGs on their lower leaves exhibited an even stronger response, with significant accumulation of ABA, SA, and JA-Ile. This suggests that OGs perception in aboveground tissues can trigger defence responses in roots. Interestingly, while OGs treatment in this study failed to confer local resistance to *B. cinerea*, it induced robust systemic resistance, which was correlated with the expression of pathogenesis-related (PR) genes. These findings underscore the complexity of OG-mediated signalling and indicate that systemic defence activation may operate through mechanisms distinct from those governing local resistance.

## OG size and modifications and mechanisms of dampening OG responses

The biological activity of OGs is influenced by their degree of polymerisation (DP), but the optimal DP required for activity remains a subject of investigation. OGs with a DP of 10–15 are widely recognised as the most effective elicitors of plant immune responses, as they stabilise the calcium-mediated “egg-box” conformation, facilitating interaction with specific receptors (Cabrera et al., 2008; De Lorenzo and Cervone, 2022). However, shorter OGs (DP 2–7) also exhibit significant biological activity in certain contexts. For example, dimeric OGs (DP 2) stimulate proteinase inhibitor production and systemic wound responses (SWR) in tomato seedlings (Moloshok et al., 1992), while OGs with DP 4–6 enhance defence-related gene expression in crops like potato and tomato and phytohormone synthesis in tobacco (Simpson et al., 1998; Montesano et al., 2001). Recently, OGs with a DP of 2–7 have been reported to act as elicitors in sugar beet, providing protection against *R. solani* (Zhao et al., 2022). Moreover, short OGs have been shown to be more effective than longer ones in delaying the onset and progression of gray mold infection on tomato fruits when applied to wounds (Lu et al., 2021). Interestingly, very short OGs (e.g., DP3) have been associated to skotomorphogenesis in etiolated seedlings (Sinclair et al., 2017). Although shorter OGs induce defence genes similarly to OGs with DP 10–15, their elicitor activity is weaker and less sustained (Davidsson et al., 2017). A more recent study (Xiao et al., 2024) shows that OGs (DP 2–7) are unable to trigger ROS production in soybean and fail to confer protection against the pathogen *Phytophthora sojae*. Moreover, pre-incubation with OGs (DP 2–7) suppresses PTI responses to PAMPs such as chitin and flg22 (Xiao et al., 2024). During fungal infection, PG activity generates immune-suppressive OGs (DP 2–7); however, plant PGIP modulates this process by promoting the formation of longer OGs (DP 10–15) with elicitor properties (Moerschbacher et al., 1999; Xiao et al., 2024).

Chemical modifications such as methyl-esterification and acetylation significantly impact OG activity. In particular, methylesterified OGs exhibit reduced elicitor potential (Jin and West, 1984; Navazio et al., 2002). The demethylesterification of HG, catalysed by pectin methylesterases (PMEs), is essential for making HG susceptible to hydrolysis by PGs, thereby enabling the production of defence-active OGs (Wolf, 2022). Changes in pectin methylation levels, which are also regulated by the activity of PMEs and PME Inhibitors, can differentially affect plant immunity depending on the pathogen’s lifestyle (specifically, its array of CWDEs) (Raiola et al., 2011; Bethke et al., 2014; Silva-Sanzana et al., 2019; Coculo and Lionetti, 2022). In wild strawberry (*Fragaria vesca*), partial de-methylation of OGs in transgenic fruit enhances resistance to *B. cinerea* (Osorio et al., 2008). A global analysis of OG composition in *A. thaliana* leaves subjected to a prolonged infection by *B. cinerea* revealed that most of the OGs remaining in the treated and macerated tissue were acetylated and methylesterified (Voxeur et al., 2019). While studies on acetylated OGs are limited, they have been shown to inhibit haustoria formation by *B. graminis* in wheat leaves (Randoux et al., 2010) and enhance protection against *A. euteiches*, a root rot pathogen in pea plants (*P. sativum*) (Selim et al., 2017). Furthermore, long, highly methyl- and acetylesterified OGs can induce the *JOX3* gene, which is involved in JA degradation, suggesting a potential role as modulators of defence responses (Voxeur et al., 2019).

Oxidation occurring at the C1 position of the reducing end and converting galacturonic acid into galactaric acid, significantly alters the biological activity of OGs (Benedetti et al., 2018). Oxidised OGs were first identified in transgenic plants expressing the “OG-machine” (Benedetti et al., 2015) after induction of this protein (Benedetti et al., 2015). The accumulation of oxidised OGs may attenuate OG-mediated signalling to prevent an excessive immune response (i.e. the hyperactivation of the plant immune system, resulting in growth inhibition, cell death, and necrosis). This regulatory mechanism ensures a balance between immune activation and normal growth processes, in line with the growth-defence trade-off model (Pontiggia et al., 2020).

The oxidation of OGs is catalysed by plant-derived OG oxidases (OGOXs), which belong to the Berberine-Bridge Enzyme-like (BBE-l) protein family. Among the 27 BBE-like proteins, at least four (OGOX1, OGOX2, OGOX3, and OGOX4) specifically oxidise OGs (Benedetti et al., 2018). These flavin adenine dinucleotide (FAD)-dependent enzymes utilise O_2_ to reoxidise the reduced FAD cofactor, producing H_2_O_2_ as a byproduct, which simultaneously regenerates their catalytic activity (Pontiggia et al., 2020; Scortica et al., 2023). Another member of the BBE-l family, named Cellodextrin Oxidase (CELLOX), oxidises cellodextrins (CD), converting the glucose at their reducing end into gluconic acid (Locci et al., 2019; Costantini et al., 2024).

Oxidised OGs exhibit reduced elicitor activity compared to their non-oxidised counterparts. Treatments with purified oxidised OGs fail to induce typical OG-triggered responses, such as ROS burst, callose deposition, and the upregulation of defence-related genes (e.g., *WRKY40, FRK1, PAD3, PGIP1, PDF1.2, PR1*) (Benedetti et al., 2018). Additionally, oxidised OGs are more resistant to fungal PG hydrolysis. Plants overexpressing OGOX enzymes show increased resistance to *B. cinerea* (Benedetti et al., 2018) but decreased resistance to *P. carotovorum*, and *P. syringae* (Salvati et al., 2024a). Notably, oxidised OGs serve as a carbon source for *P. carotovorum* and *P. syringae* but not for the fungus *B. cinerea*. Kinetic studies and molecular simulations indicate that penta-galacturonic acid is the preferred substrate for OGOX1, while shorter oligomers exhibit limited oxidation (Frezzini et al., 2023).

The H_2_O_2_ produced by OGOX enzymes may contribute to various physiological processes and have signalling activity. For instance, extracellular peroxidases (PODs) utilise this H_2_O_2_ to reinforce the cell wall via lignification and to oxidise auxin, thereby inhibiting plant growth during pathogen attacks (Scortica et al., 2022). Additionally, OGOX1 has been shown to scavenge cation radicals, particularly during the oxidation of short OGs (DP ≤ 4), highlighting its role in mitigating oxidative stress during advanced infection stages (Scortica et al., 2023).

Finally, as discussed earlier, OGs modifications such as oxidation and methylation may hamper disrupt the ability OGs to bind RALF23 and RALF1 peptides, which regulate FERONIA-LLG1-mediated endocytosis (Liu et al., 2024). Transient overexpression of OGOX1 and PMEI in *Nicotiana benthamiana* leaves prevents the activation of this endocytic pathway, triggered by RALF-OGs condensates (Liu et al., 2024). Although the precise connection between reduced elicitor activity of oxidised OGs and inhibition of endocytosis remains to be elucidated, these processes may be linked.

The activity of OGs depends on their structure, but understanding how they are recognised by plants is still a challenge. Studying pectic fragments *in vivo* is complex because plant CW is dynamic and constantly changing. The extraction and characterisation of OGs is influenced by several factors, such as the extraction method used, the plant’s physiological state, and the timing of sample collection. These variables make it difficult to link a specific OG fragment to a clear plant response (Benedetti et al., 2015; Pontiggia et al., 2015; Benedetti et al., 2017; Voxeur et al., 2019; Liu et al., 2023).

## OGs for a sustainable and eco-friendly agriculture

**Figure 2:**
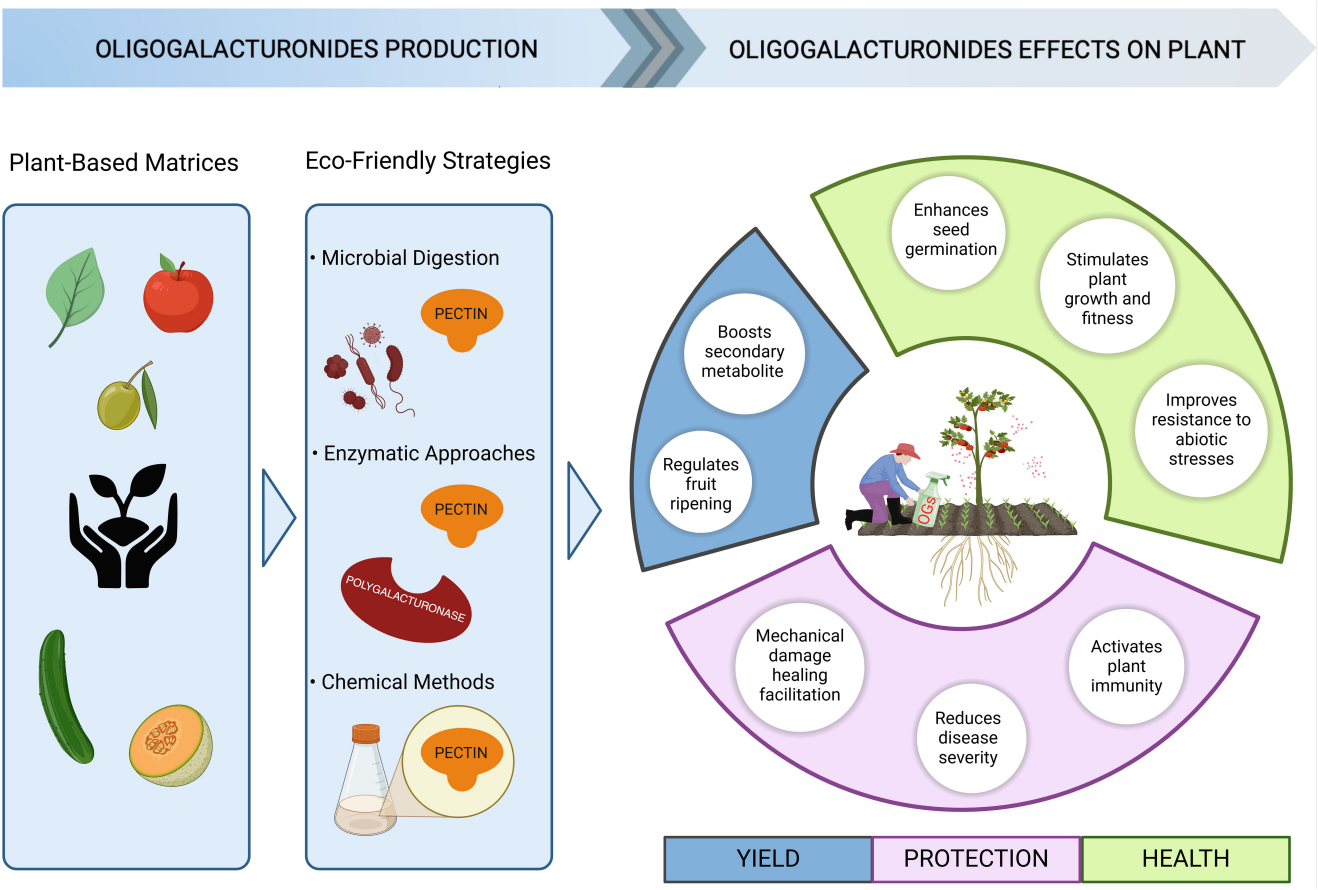
Benefits of oligogalacturonides use for sustainable agriculture. OGs are valuable molecules in agriculture due to their sustainability in production, minimal environmental impact, and beneficial effects on plants. OG-based products could be produced starting from the degradation of plant-based matrices that are rich in polysaccharides through different processes, such as microbial digestion, enzymatic hydrolysis, or safe chemical treatment. The incorporation of agricultural waste within a circular economy framework not only promotes sustainability but also reduces environmental impact and production costs. The application of OG-based products not only induces plant resistance against biotic and abiotic stress but also improves plant yield and fitness, affecting different processes as reported in the right circle (created in BioRender).

One of the most pressing challenges in the coming years is addressing the significant impact of plant diseases, which are responsible for an estimated 40% annual loss in the yield of key food crops worldwide. This substantial reduction not only threatens global food security but also undermines efforts to meet the growing demand for agricultural production driven by population growth (Wang et al., 2024). Moreover, the increased intensity and frequency of abiotic stresses due to climate change further alter the plant susceptibility to pests and pathogens (Leisner et al., 2022). Presently, the control of diseases and insect pests primarily depends on chemical products, which are associated to health risks and environmental hazards besides being expensive, making it necessary alternative crop protection methods (Flors et al., 2024). Is important, indeed, to develop new strategies to enhance plant immunity, one of which is enhance natural plant resistance through genetic approaches (Wang et al., 2024). However, the improved resistance can result in decreased growth and yield according to the growth-defence trade-off (Figueroa-Macías et al., 2021). Thus, understanding the signalling pathways related to plant immunity and defence responses is essential for develop new biotechnological strategies aimed at reducing losses from pathogens without compromising yield and favour a more sustainable agriculture. The use of CW-DAMPs including OGs could be a viable alternative to conventional agrochemicals, as they are eco-friendly and not harmful to the environment (**Figure 2**). OGs represent a promising class of natural biopesticides due to their bioactive properties as plant immunostimulants enhancing resistance against different classes of phytopathogens and in different plant species. According to their classification as “low-risk active substances” through the EU Regulation (EC) No 1107/2009, OGs are widely used as natural plant protection products (Salvati et al., 2024b) in line with the EU intent of reduce the use and the risk of chemical pesticides by 50% for 2030 (Eurostat, 2023). Indeed, as already reviewed increasing number of evidence demonstrated how OGs can improve plant resistance against pathogens in several plant species of agronomic interest grown in controlled conditions.

Although their use as primary ingredients in commercial products is still limited compared to other biostimulants o biopesticides, several companies developed and commercialised product with OGs in their formulation and in 2018 for the first time a OG-based product, the FytoSave^®^ obtain the phytosanitary registration for use in organic agriculture (Guarnizo et al., 2020). FytoSave^®^ and some similar products such as FytoSol^®^, is based on the COS-OGA formulation which combines chitosan oligomers (COS) with pectin-derived oligogalacturonides simulating the natural interaction between plants and fungal pathogens (van Aubel et al., 2018). Under greenhouse conditions, repetitive foliar spraying of the COS-OGA mixture on tomato plants confers effective systemic resistance against powdery mildew (*Leveillula taurica*) in a cumulative manner depending on the number of provided treatments (van Aubel et al., 2016). Commercial COS-OGA formulations are also effective against the root-knot nematode *Meloidogyne graminicola* once sprayed in rice leaves (Singh et al., 2019) and a similar effect was also demonstrated in potato and tomato against *Phytophthora infestans* and *Alternaria solani* respectively after repeated preventive treatments (Clinckemaillie et al., 2017; van Aubel et al., 2018; Bektas, 2022). The effectiveness of these commercial products, in managing *Colletotrichum acutatum* infection was evaluated on two olive cultivars, Koroneiki and Kalamon. While Fytosave^®^ showed no significant effect on Koroneiki, it notably reduced disease severity and conidia production on Kalamon drupes. The experiments highlight variations in product efficacy across different cultivars and infection conditions (Varveri et al., 2024). Although most studies on OGs and OG-based formulations were conducted under controlled conditions, in recent years, there has been an increasing number of experiments performed in field under natural conditions. Tests conducted in different European states (Spain, France and Belgium) under commercial growing conditions, showed that the COS-OGA mixture has high effectiveness against natural infection of powdery mildew on grapevine and cucumber plants with results comparable to those obtained with chemical sulfur treatments (van Aubel et al., 2014). Similarly, three-year studies conducted in Italian vineyards of *Vitis vinifera* ‘Barbera’ the application of COS-OGA formula alone or in combination with copper and sulfur at low doses enhanced the resistance against powdery and downy mildew (Taibi et al., 2023, 2024), protection confirmed also in another study conducted in “Nebbiolo” vineyards (Rantsiou et al., 2020).

The efficacy of OG treatment to prevent disease in plants grown under natural conditions, is also demonstrated with spray application of Planticine^®^ which improves not only the resistance against fungi but also the aesthetic and functional values of turf grasses (Radkowski et al., 2024). Planticine^®^ (INTERMAG Sp. z o.o.) is a biodegradable, non-toxic, and water-soluble commercial product characterised by OGs as main component able to induce plant resistance against microbial pathogens and herbivores as effectively demonstrated in tomato plants and cucumbers (Rakoczy-Lelek et al., 2023). Planticine^®^ act as elicitor reducing powdery mildew and trips infestation (Rakoczy-Lelek et al., 2023).

Although few studies directly compare the effectiveness of OG-based products with conventional chemical pesticides, some experimental evidence sheds light on their mechanism of action. In tomato plants infected with powdery mildew, COS-OGA demonstrated a comparable ability to reduce disease severity, though it was slightly less effective at preventing disease incidence compared to the chemical fungicide Imazalil (van Aubel et al., 2016). Nevertheless, plants treated with COS-OGA exhibited significantly better protection than those in the untreated control group, underscoring its potential as a sustainable alternative to chemical treatments (van Aubel et al., 2016).

In grapevine trials, 100% of untreated bunches were infected with powdery mildew, whereas sulfur-based fungicides significantly reduced disease incidence to 21.5%. While OG-based products were less effective in preventing disease spread, with 67% of grape bunches showing symptoms, they nonetheless reduced disease severity to a level comparable to the chemical treatment (van Aubel et al., 2014). This pattern was consistent across trials conducted in France, Spain, and Belgium (van Aubel et al., 2014). Further studies in vineyards support the potential of COS-OGA as part of an integrated pest management strategy. In control conditions, 58% of grape clusters were infected by *E. necator* with a disease severity of 8%. Both standard pesticide treatments and COS-OGA applications achieved comparable reductions in disease incidence (<30%) and severity (<5%) (Rantsiou et al., 2020).

In turfgrass, treatments with PLANTICINE^®^ significantly enhanced visual quality, improving overall appearance and canopy index. Additionally, it effectively reduced fungal diseases such as pink snow mold and leaf spot. However, while commercial fungicides offered greater control over fungal pathogens, they came with a higher environmental cost. Soil quality analysis indicated that PLANTICINE^®^ had a less detrimental impact on soil characteristics, highlighting its potential as a more sustainable option for turfgrass cultivation (Radkowski et al., 2024). This highlights how OG-based treatments can be incorporated into pest management strategies, providing an environmentally friendly alternative to conventional fungicides while maintaining effective disease control.

The key difference between the action of chemical pesticides and OG-based products lies in their role as plant elicitors. Unlike traditional fungicides, which directly impair life cycle or vitality of pathogens, OG-based products activate the plant’s natural defence mechanisms. This stimulation helps restrict disease spread, slow its progression, reduce the affected plant area, and limit fungal sporulation (Clinckemaillie et al., 2017). Therefore, it is clear that OG-based treatments are less effective at preventing initial infection but excel in limiting its spread within affected tissues (van Aubel et al., 2016). This mode of action makes COS-OGA a valuable tool for slowing disease progression (Clinckemaillie et al., 2017).

Although further studies are necessary to fully evaluate the effects of OG-based formulations on crops in natural environmental conditions, current experimental findings suggest that these products could serve as a promising alternative to conventional pesticides. Their ability to enhance natural defence mechanisms aligns with the goals of sustainable agriculture, supporting reduced pesticide use and greater environmental protection.

This class of elicitors not only influences resistance but also growth and plant fitness. Interestingly, in sugar beet, OGs significantly improve seedlings germination when used as seed pretreatment (Zhao et al., 2022). In bean, the use of Pectimorf^®^, a phyto-elicitor with OGs derived from citrus industry by-products as main component (Reyes-Perez et al., 2022), increases nitrogen fixation and consequently nodulation and plant growth (Suarez et al., 2013; Lara-Acosta et al., 2019, 2023). Moreover, the simultaneous application of Pectimorf^®^ and arbuscular mycorrhizal fungal (AMF) strains to *Coffea arabica* L. seedlings enhances their growth during the acclimatation phase (Gonzàlez-Vega et al., 2023) and a similar effect in response to the product application were also reported in sugarcane, banana and cocoa plants (Izquierdo et al., 2009; Reyes-Perez et al., 2022; Gonzàlez-Vega et al., 2023).

In rice and tomato the presence of OGs in the soil protect plants from abiotic stress conditions, such as salt stress and heavy metal contamination (Cartaya-Rubio et al., 2016; Cartaya et al., 2017; Acosta et al., 2018; Núñez-Vázquez et al., 2018; Cartaya-Rubio et al., 2024). Interestingly, the application of OGs enhanced the Aluminium (Al)-induced expression regulation of two soybean SAUR-like genes and this effect is prevented by the overexpression of berberine-bridge protein GmBBE-like43, suggesting that the inactivation of OGs in cell walls mediated by GmBBE-like43 contributes to soybean’s Al-tolerance mechanism (Chen et al., 2022). Furthermore, in response to cold stress an upregulation of WAK genes was observed in rice and in a cold-tolerant maize genotype suggesting the involvement of WAKs and consequently of OGs in cold-induced responses (Zhang et al., 2012). OGs can also influence the wound-healing capacity in plants. Specifically, in tomato plants, OGs accelerate wound healing by increasing callose deposition (Lu et al., 2021). Moreover, in Goji berry, it has been observed that they OGs can reduce the damage caused by wounding, minimising the formation of ROS through the stimulation of superoxide dismutase (SOD) activity (Pei et al., 2024).

Several studies showed to that OGs have a role as regulator of fruit ripening processes reducing fruit softening as demonstrated in goji berries, grapevine, tomato and strawberry (Osorio et al., 2011; Ma et al., 2016; Paniagua et al., 2020; Rantsiou et al., 2020; Yang et al., 2022; Pei et al., 2024). Therefore, exogenous treatment with OGs can improve the shelf life of different fruit varieties and reduce postharvest losses caused by mechanical damage incurred during shipping and handling, maintaining fruit firmness and preventing pathogens infections (Ma et al., 2016; Paniagua et al., 2020; Rantsiou et al., 2020; Yang et al., 2022; Pei et al., 2024). Fruit ripening is a complex process regulated by enzymes like plant PGs that degrade the pectin layer of the cell wall. While it is known that plant PGIPs regulate fungal PG activity during infections by forming a protein complex for bioactive OG accumulation (Xiao et al., 2024), a similar mechanism in fruit ripening is still unclear. However, recently, a model involving PG-PGIP interactions in *Rubus idaeus* suggests that also among endogenous plant proteins a similar albeit non identical interaction could also occur during fruit ripening driving the formation of OGs with regulatory physiological function (Morales-Quintana et al., 2023).

However, although OGs and OG-based products are considered “low-risk active substances” for environment, their application on crops may cause secondary effects (Martínez-Gómez et al., 2023). If on one hand OG treatment can influence the plant secondary metabolism leading to the accumulation of healthy compound such as stilbene derivatives including resveratrol, well-known to lowering the risk of heart disease and atherosclerosis (van Aubel et al., 2014), on the other one, the perturbation of hormonal signalling and the induced metabolic changes involved in fruit ripening (Pott et al., 2020) could compromise the organoleptic characteristic of the fruit and their derivatives product such as reported for wine produced from OG-treated grapevines (Rantsiou et al., 2020).

Given the enormous application potential in agriculture, numerous companies are developing innovative solutions to produce CW-DAMPs-based products using plant-based matrices rich in polysaccharides, including agricultural waste within a circular economy framework. These approaches not only support sustainability but also help reduce environmental impact and production costs, making biostimulants more accessible and aligned with the needs of modern, eco-friendly agriculture. For example, as rich source of pectin, citrus peel wastes (CPWs) (lemon, orange and mandarin) can be efficiently used for the extraction of pectin-derived compounds including OGs with different DP and chemical modification (Gómez et al., 2016; Yang et al., 2020; Morales et al., 2023), but an increasing number of studies highlight the possibility to produced bioactive and plant protective OGs starting from different substrates such as olive pomace and mill wastewaters (Greco et al., 2024; Salvati et al., 2024b), sugar beet pulp (Pińkowska et al., 2021; Carton et al., 2024; Martínez-Gómez et al., 2024), melon peels (Rico et al., 2021, 2022), apple (Cano et al., 2021) cucumber juice (Zhang et al., 2024) or directly from commercial polygalacturonic acid and pectin (Chen et al., 2013; Valdivieso Ramirez et al., 2021; Yang et al., 2021b).

Different strategies can be used for the degradation of pectin and pectin rich biomasses such as by microbial digestion (Yang et al., 2020, 2021), chemical methods (acid hydrolysis, hydrothermal treatments, eco-friendly sodium acetate/urea [NaOAc:urea:water) deep eutectic solvent (DES)] (Grohmann et al., 1998; Martínez et al., 2010; Chen et al., 2013; Gómez et al., 2013, 2016; Pińkowska et al., 2021; Rico et al., 2021; Valdivieso Ramirez et al., 2021; Rico et al., 2022; Shao et al., 2022; Morales et al., 2023; Carton et al., 2024; Martínez-Gómez et al., 2024), enzymatic approaches (Martínez et al., 2009; Combo et al., 2012; Martínez Sabajanes et al., 2012) or a combination of both (Qian et al., 2024). The utilisation of plant-based matrices and agricultural waste to produce CW-DAMPs-based biostimulants offers a promising path toward sustainable agriculture. By employing various degradation strategies, these approaches not only enhance resource efficiency but also contribute to eco-friendly practices, reducing both environmental impact and production costs.

## Concluding remarks

The plant CW is a cornerstone of plant defence, acting as a primary physical and chemical barrier against pathogens. Central to this defence is the release of CW-DAMPs, such as OGs, which are generated from the degradation of pectins during cellular damage or microbial attack. These bioactive molecules serve as essential signals that activate intricate pathways, orchestrating immune responses and integrating with hormonal networks. This complex signalling system fine-tunes defence mechanisms against biotic and abiotic stresses while preventing overactive immune responses that could compromise plant fitness.

Despite how OGs are detected and how they trigger signals in plants is not fully understood, their biotechnological potential is undeniable. Studying how OGs interact with plants and microbes can lead to new strategies for protecting and improving crops. Research has shown that OGs can enhance plant immunity, offering a sustainable alternative to chemical pesticides and minimising environmental impact. Additionally, producing OGs from plant materials follows circular economic principles, reducing costs, minimising waste, and lowering environmental impact.

In conclusion, the “case study of oligogalacturonides” demonstrates how deciphering the perception and signalling pathways of bioactive molecules such as CW-DAMPs can transform strategies for crop protection and agricultural sustainability.
